# RESULTS OF SURGICAL TREATMENT OF CARPAL TUNNEL SYNDROME USING TWO VARIATIONS OF OPEN TECHNIQUE

**DOI:** 10.1590/1413-785220233102e260893

**Published:** 2023-05-01

**Authors:** PATRÍCIA MARIA DE MORAES BARROS FUCS, YUSSEF ALI ABDOUNI, ANA CAROLINA DA SILVA LOVATO

**Affiliations:** 1 Santa Casa de Misericórdia de Sao Paulo, Faculdade de Ciências Médicas, Departamento de Ortopedia e Traumatologia, Sao Paulo, SP, Brazil.

**Keywords:** Carpal Tunnel Syndrome, Median Nerve, Median Neuropathy, Síndrome do Túnel do Carpo, Nervo Mediano, Neuropatia Mediana

## Abstract

Transverse carpal ligament (TCL) opening is the treatment of choice for carpal tunnel syndrome. However, complications such as loss of grip strength and anterior displacement of the median nerve are described as common complications associated with this technique. Thus, techniques that reconstruct or extend TCL are described to reduce the incidence of these complications. Objective: To evaluate the effectiveness of TCL enlargement through Z-plasty and the reduction of complications by comparing it with the complete opening of the ligament. Materials and Methods: A prospective and randomized intervention study was conducted in 56 patients. They were divided into 2 groups: 1) complete opening of TCL 2) TCL enlargement via Z-plasty. We evaluated grip strength, sensitivity, and functional evaluation using the QuickDASH questionnaire. Results: There was no statistically significant difference in the improvement of scores with QuickDASH between the two techniques. The sensitivity test was better in patients subjected to TCL enlargement, whereas grip strength increased in the group subjected to complete TCL opening. Conclusion: According to the results of this study, the complete opening of the TCL showed no reduction in grip strength, although it showed inferior recovery to postoperative sensitivity. Both techniques were equivalent in functional improvement. Thus, Z-plasty showed no identifiable benefits for TCL enlargement. ***Level of Evidence III, Randomized Clinical Trial.***

## RESUMO

A abertura completa do ligamento transverso do carpo (LTC) é o tratamento de escolha para a síndrome do túnel do carpo. No entanto, complicações como perda de força de preensão e deslocamento anterior do nervo mediano são complicações comuns associadas a essa técnica. Assim, descrevem-se técnicas que reconstroem ou alargam o LTC visando reduzir a incidência dessas complicações. Objetivo: Avaliar a efetividade do alargamento do LTC através de Z-plastia e a diminuição das complicações, comparando a técnica com a abertura completa do ligamento. Métodos: Realizou-se um estudo de intervenção, prospectivo e randomizado com 56 pacientes. Estes foram divididos em dois grupos: 1) abertura completa do LTC e 2) alargamento do LTC através de Z-plastia. Avaliamos força de preensão e sensibilidade e realizamos avalição funcional por meio do questionário *Quick Disabilities of Arm, Shoulder and Hand* (QuickDASH). Resultados: Não houve diferença estatisticamente significante na melhora dos escores entre as duas técnicas. O teste de sensibilidade teve melhores resultados nos pacientes submetidos ao alargamento do LTC, enquanto a força de preensão teve maior acréscimo no grupo submetido à abertura completa do LTC. Conclusão: A abertura completa do LTC não levou à redução da força de preensão, apesar de ter se mostrado inferior na recuperação da sensibilidade no pós-operatório. As duas técnicas foram equivalentes na melhora funcional. Dessa forma, não encontramos benefícios identificáveis na realização da Z-plastia para alargamento do LTC. **Nível de Evidência III, Ensaio Clínico Randomizado.**

## INTRODUCTION

Carpal tunnel syndrome is the most common compressive neuropathy of the upper limbs, affecting 4% to 5% of the general population.^1^ ^), (^ ^2^ Women are more affected than men, it is more prevalent in the 40 to 60 years age range, and is usually bilateral.^3^ Risk factors for the disease include obesity, repetitive wrist movements, pregnancy, family history, and rheumatoid arthritis. ^(^ ^4^

Conservative methods can be used for treatment and the most common is the use of orthotic devices and local injection of corticosteroids. ^(^ ^5^ ^), (^ ^6^ More recently, exercises involving nerve excursion showed a reduction in the number of surgical interventions. ^(^ ^7^ Nerve excursion can be an option to speed up functional recovery. ^(^ ^8^ Other methods such as platelet-rich plasma injections^9^ and shockwave therapy^10^ are being studied as options to treat this disease, but still lack data to prove their effectiveness.

The literature lacks consensus on the best time for surgical indication, but studies show that surgery is generally more effective than conservative treatment in terms of recurrence rate, improvement of symptoms, and hand function. ^(^ ^11^ However, the best time for surgical treatment should be discussed with the patient as their symptoms are not always directly related to the findings of physical examination and electroneuromyography. ^(^ ^12^

Conservative treatment generates positive responses in 80% of patients. Relapse rates of symptoms after conservative treatment range from 8% to 80%.^12^ ^)- (^ ^14^ Surgical treatment can be performed by an open approach or endoscopically. Similar results and complication rates are observed in both techniques. ^(^ ^15^ ^), (^ ^16^

Regarding surgical treatment, the complete opening of the transverse carpal ligament (TCL) remains a treatment of choice in refractory cases. As common complications associated with this technique, we can mention the reduction of grip strength and pain on the thenar and hypothenar eminence, also called “pillar pain.” ^(^ ^17^ Discomfort at the site of the scar is also a common complication, with incidence rates ranging from 19% to 61%.^13^

The reduction in grip strength is attributed by several authors to the loss of the pulley effect of the TCL on the flexor tendons. ^(^ ^15^ To avoid such a complication, several techniques of TCL reconstruction were postulated after its complete section, ^(^ ^16^ as well as techniques that only widen the ligament without completely sectioning it. ^(^ ^17^ Another complication described is the subluxation of the median nerve, observed intraoperatively, in 60% of the patients reoperated for recurrence of carpal tunnel syndrome. ^(^ ^2^ To avoid these effects and maintain tendons and nerves within the TCL, some authors use Z-plasty for TCL enlargement and reconstruction. ^(^ ^6^ ^), (^ ^8^

This study aims to compare the results of grip strength, symptom improvement, and postoperative sensitivity of the technique of TCL widening by Z-plasty with the complete opening for the treatment of patients with carpal tunnel syndrome.

## METHODS

### Population studied

In total, 56 patients were evaluated, 20 men and 36 women, with clinical criteria for the disease in a prospective randomized intervention study. The Tinel’s sign and the Durkan and Phalen tests were evaluated, as well as thenar atrophy, grip strength, and complaints of paresthesia in the median nerve. The electroneuromyography study was not used in the diagnostic criteria. Studies show that this method has limitations, including the inability to predict what patients will benefit most from surgery or conservative treatment. ^(^ ^7^

Patients with complaints of paresthesia and pain in the ulnar nerve path and patients with advanced thenar atrophy and loss of opposability were excluded; patients with signs of high compression of the median, associated diseases such as rheumatoid arthritis and other connective tissue diseases, malignancies, renal diseases, distal radius fractures, congenital neuropathies, spinal diseases, diabetes mellitus, fibromyalgia, pregnant women, or thyroid diseases were also excluded. Patients previously operated on for median nerve decompression (reoperation) were also excluded.

This study was approved by the Research Ethics Committee of the School of Medical Sciences of Santa Casa de São Paulo under opinion No. 4985725.

### Evaluation criteria

Patients who met the inclusion criteria of the study completed the informed consent form after the appropriate explanations. All data evaluated regarded the side to be operated on.

The patients completed the Quick Disabilities of the Arm, Shoulder and Hand Outcome Measure (QuickDASH) questionnaire, composed of eleven items that address symptoms and skills of daily living in people with any or several disorders involving the upper limb, with the advantage of providing the same quality of information with fewer items for the patient to complete, facilitating scoring for the clinician or researcher. Like the regular DASH, it provides a scale with scores of 0-100 points, with 100 indicating the highest deficiency. QuickDASH is comparable to regular DASH, and its construct validity and sensitivity suggest that QuickDASH scores should observe disabilities and symptoms that are relatively similar to those predicted by the full version. ^(^ ^2^

The patients were also subjected to the sensitivity test of the median nerve by the Semmes-Weinstein monofilaments. Six monofilaments (pocket model - “Sensikit”) of nylons number 612, 38 mm in length, and different diameters that exert a specific force in the tested area corresponding to weight variation from 0.05 to 300 g was used. For filling out the disability degree form, the parameters used were those by the MS to record the degree of disability, in which the perception of monofilaments of 0.05 g (green), 0.2 g (blue), and 2.0 g (violet) indicate degree 0 of disability; whereas the non-perception of the monofilament of 2.0 g (violet) and the perception or not of the other monofilaments (4.0 g, 10.0 g, and 300.0 g) indicate grade I disability.

The grip strength was evaluated according to the recommendation of the American Society of Hand Therapists (ASHT), by using the Jamar dynamometer^®^, in the second position (of five), referring to the size of the handle. The patients remained seated in an office chair (without arms) with their spine erect, keeping the knee flexion angle at 90°, the shoulder positioned in adduction and neutral rotation, the elbow flexed at 90°, with their forearms in half pronation and neutral wrist, which could be moved up to 30° of extension. Their arm was kept suspended in the air with their hand positioned on the dynamometer, which was supported by the evaluator. Grip strength measured by the dynamometer does not reflect all the situations encountered in the gestures of everyday life or at work. Therefore, this parameter was not used isolated in the evaluation of the results.

### Follow-up

Patients were evaluated in the immediate preoperative period and with 30, 60, and 180 postoperative days. All patients were evaluated by the same examiner on all dates and criteria. Patients who could not be reevaluated at any of these intervals were excluded.

### Surgical method

The patients were randomly divided into two groups: those subjected to the “open” method and another group to the “zetaplasty” method.

The procedure was performed in a surgical environment, always by the same surgeon, and the type of anesthesia was determined by the anesthesiologist. The same classical volar access route to the TCL was performed in all patients, being an incision of approximately 3 cm, slightly ulnar to the thenar flexion fold. The TCL was cross-sectioned in the “open” group, and by zetaplasty in the group of the same name.

**Figure 1:**
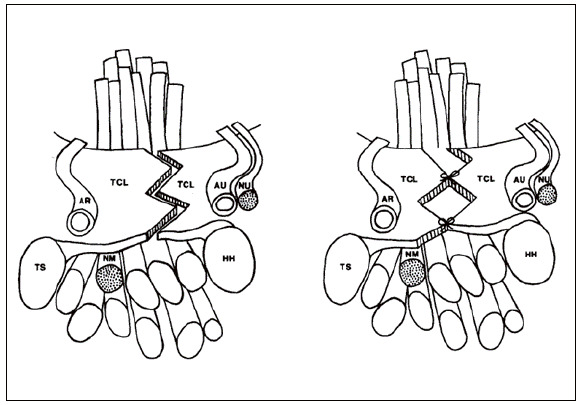
TCL incision in patients in the group subjected to TCL enlargement and reconstruction by Z-plasty. TCL: transverse carpal ligament; TS: scaphoid tuberosity; HH: hamate hamulus; RA: radial artery, UA: ulnar artery; UN: ulnar nerve; MN: median nerve.

Source: Karlsson et al. ^(^ ^12^

The complete release of the TCL was verified, being left open in patients of the open group. In the “zetaplasty” group, the TCL was sutured so as not to cause tension or new nerve compression, together with the tips of the “Z” formed by the incision, as Figure 1 exemplifies.

All patients underwent early mobilization and occupational therapy in the postoperative period. Plastered immobilization or orthotic devices were not used in any case in the postoperative period since their use showed no beneficial effect when compared to simple compressive dressing. Moreover, this practice increases the total surgical time and can thus be safely abandoned. ^(^ ^5^

## RESULTS

The statistical analysis used the Student’s *t*-test. The quantitative variables are described by their mean and 95% confidence interval for those considered normal. The two-sample *t*-test was used to compare whether the proportion of responses of two certain variables and/or their levels is statistically significant.

Our objective was to compare the results between the two types of surgery. We started with the mean of DASH and Jam in each of the four periods. We used the Student’s *t-*test.

- MF: Semmes-Weinstein monofilament test
- JAM: Jamar dynamometer^®^

**Figure 2:**
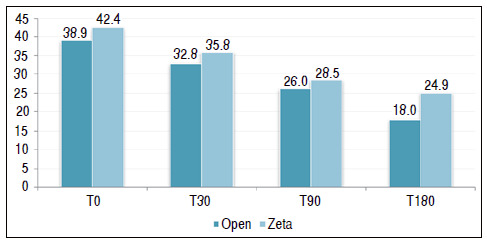
Comparison between the types of surgery in relation to functional recovery evaluated by the Quick Disabilities of Arm, Shoulder and Hand by follow-up time.

**Figure 3:**
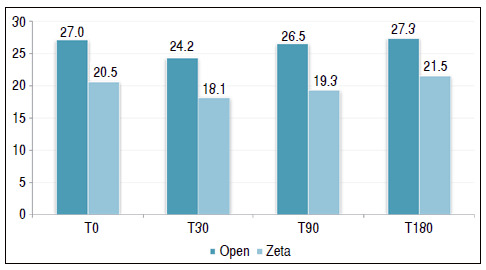
Comparison between the types of surgery in relation to the grip strength measured by the Jamar dynamometer by follow-up time.

Table 1 and Figure 2 show a comparison between the two surgical techniques and their outcome using the QuickDASH questionnaire as a parameter. Table 2 and Figure 3 use the grip strength measured by Jamar^®^ to compare the two techniques.

**Table 1:** Comparison between the types of surgery in relation to functional recovery evaluated by the Quick Disabilities of Arm, Shoulder and Hand by follow-up time.

DASH		Mean	Median	Standard Deviation	VC	Min	Max	N	CI	p-value
T0	Open	38.9	35	19.9	51%	10	80	28	7.4	0.483
	Zeta	42.4	45	16.4	39%	15	80	27	6.2	
T30	Open	32.8	30.5	21.7	66%	7	75	28	8.0	0.592
	Zeta	35.8	35	20.4	57%	5	70	27	7.7	
T90	Open	26.0	20.5	19.7	76%	2	74	28	7.3	0.662
	Zeta	28.5	20	22.1	77%	4	73	27	8.3	
T180	Open	18.0	13.5	16.6	92%	0	58	28	6.1	0.193
	Zeta	24.9	17	21.5	87%	1	77	27	8.1	

**Table 2:** Comparison between the types of surgery in relation to the grip strength measured by the Jamar dynamometer by follow-up time.

Jam		Mean	Median	Standard Deviation	VC	Min	Max	N	CI	p-value
T0	Open	27.0	26	9.0	33%	12	44	28	3.3	0.002
	Zeta	20.5	18	4.9	24%	12	30	27	1.8	
T30	Open	24.2	22	9.3	38%	8	40	28	3.4	0.003
	Zeta	18.1	18	4.8	27%	12	30	27	1.8	
T90	Open	26.5	27	8.9	33%	10	40	28	3.3	<0.001
	Zeta	19.3	18	4.5	23%	10	28	27	1.7	
T180	Open	27.3	27	8.9	33%	10	40	28	3.3	0.004

When QuickDASH was evaluated as an isolated parameter, the types of surgeries, in all four follow-up moments, showed no statistically significant mean difference. Grip strength showed a mean difference between the types of surgery at all times of the follow-up. The mean was always higher in the group subjected to complete opening of the TCL compared with the Z-plasty group. The largest difference occurred in the T90, in which the mean of open surgery was 26.5 versus 19.3 of the Z-plasty group (p < 0.001).

**Figure 4:**
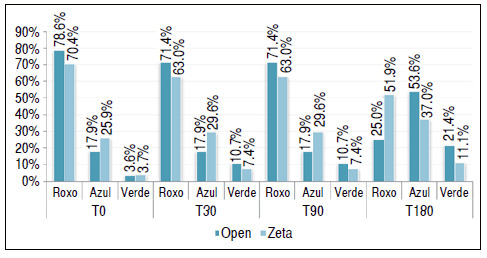
Comparison between the types of surgery in relation to the grip strength measured by the Jamar dynamometer by follow-up time.

In Table 3 and Figure 4, we compared the surgeries for the distribution of the relative frequency of the results of the sensitivity test using the monofilaments, in which we used the two-sample *t*-test.

**Table 3:** Comparison between the types of surgery in relation to the grip strength measured by the Jamar dynamometer by follow-up time.

MF N		Open		Zeta		p-value
		%	N	%		
T0	Violet	22	78.6	19	70.4	0.485
	Blue	5	17.9	7	25.9	0.469
	Green	1	3.6	1	3.7	0.979
T30	Violet	20	71.4	17	63	0.504
	Blue	5	17.9	8	29.6	0.304
	Green	3	10.7	2	7.4	0.670
T90	Violet	20	71.4	17	63	0.504
	Blue	5	17.9	8	29.6	0.304
	Green	3	10.7	2	7.4	0.670
T180	Violet	7	25.0	14	51.9	0.040
	Blue	15	53.6	10	37.0	0.218
	Green	16	21.4	3	11.1	0.301

We found that there is only a statistically significant difference between the types of surgery in the distribution of the violet result in T180, in which in the open surgery group the index was 25.0% and in the Z-plasty was 51.9% (p = 0.040).

**Figure 5:**
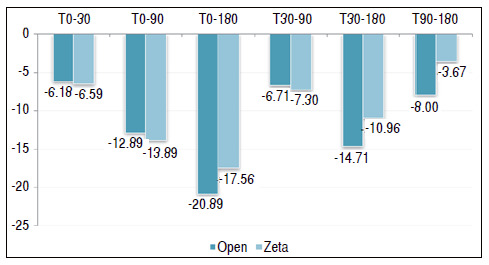
Comparison between the types of surgery for delta values of the Disabilities of Arm, Shoulder and Hand, in which: a positive value indicates functional improvement and a negative value indicates functional decrease.

**Figure 6:**
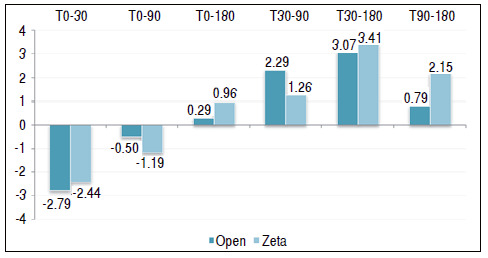
Comparison between the types of surgery for delta grip strength measured by the Jamar dynamometer, in which: a positive value indicates improvement in grip strength and a negative value indicates a decrease in it.

The gain/delta was also performed between the moments for QuickDASH and grip strength, whose results are shown in Table 4 and Figure 5 and Table 5 and Figure 6, respectively. This gain is simply the simple mathematical difference between the times, in which a positive value indicates an increase in the value between the times involved and a negative value indicates a reduction. We reused the Student’s *t*-test to compare the types of surgery as to the mean of the QuickDASH deltas and grip strength.

**Table 4:** Comparison between the types of surgery for delta values of the Disabilities of Arm, Shoulder and Hand, in which: a positive value indicates functional improvement and a negative value indicates functional decrease.

DASH delta		Mean	Median	Standard Deviation	VC	Min	Max	N	CI	p-value
T0-30	Open	- 6.18	- 2.5	11.95	193%	- 38	15	28	4.43	0.900
	Zeta	- 6.59	- 5	12.42	188%	- 27	15	27	4.68	
T0-90	Open	- 12.89	- 13.5	14.02	109%	- 43	17	28	5.19	0.808
	Zeta	- 13.89	- 15	16.21	117%	- 48	18	27	6.12	
T0-180	Open	- 20.89	- 17	15.76	75%	- 55	20	28	5.84	0.439
	Zeta	- 17.56	- 15	15.96	91%	- 48	22	27	6.02	
T30-90	Open	- 6.71	- 5	8.33	124%	- 29	5	28	3.08	0.810
	Zeta	- 7.30	- 5	9.47	130%	- 31	6	27	3.57	
T30-180	Open	- 14.71	- 11.5	11.66	79%	- 40	5	28	4.32	0.233
	Zeta	- 10.96	- 11	11.42	104%	- 33	8	27	4.31	
T90-180	Open	- 8.00	- 6.5	8.07	101%	- 30	3	28	2.99	0.030
	Zeta	- 3.67	- 3	6.16	168%	- 21	5	27	2.32	

**Table 5:** Comparison between the types of surgery for delta grip strength measured by the Jamar dynamometer, in which: a positive value indicates improvement in grip strength and a negative value indicates a decrease in it.

Jam delta		Mean	Median	Standard Deviation	VC	Min	Max	N	CI	p-value
T0-30	Open	- 2.79	- 2	3.78	136%	- 12	4	28	1.40	0.722
	Zeta	- 2	- 2	3.25	133%	- 8	2	27	1.23	
T0-90	Open	- 0.50	0	3.95	790%	- 8	8	28	1.46	0.509
	Zeta	- 1.19	0	3.69	311%	- 10	4	27	1.39	
T0-180	Open	0.29	0	4.54	1591%	- 10	10	28	1.68	0.531
	Zeta	0.96	2	3.30	343%	- 6	8	27	1.24	
T30-90	Open	2.29	2	3.21	140%	- 2	8	28	1.19	0.229
	Zeta	1.26	0	3.05	242%	- 2	8	27	1.15	
T30-180	Open	3.07	2	4.27	139%	- 6	14	28	1.58	0.745
	Zeta	3.41	4	3.27	96%	- 2	12	27	1.23	
T90-180	Open	0.79	0	2.39	305%	- 4	6	28	0.89	0.051
	Zeta	2.15	2	2.66	124%	- 2	8	27	1.00	

In the gain/delta analysis, we found only a mean difference between the types of surgery when we used QuickDASH in the T90-180 delta, with reduced values in both surgeries. For open surgery, the mean was −8.00 versus −3.67 in the Z-plasty group (p = 0.030).

**Figure 7:**
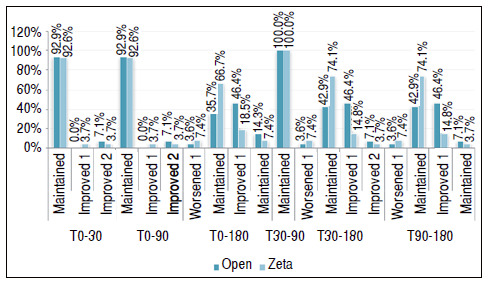
Comparison between the types of surgery for delta recovery of sensitivity measured by monofilaments, in which: a positive value indicates improvement in sensitivity and a negative value indicates its decrease.

Finally, Table 6 and Figure 7 show a comparison between the types of surgery for the Delta in the sensitivity parameter. The two-sample *t*-test equality test was used.

**Table 6:** Comparison between the types of surgery for delta recovery of sensitivity measured by monofilaments, in which: a positive value indicates improvement in sensitivity and a negative value indicates its decrease.

MF delta		Open		Zeta		p-value
		N	%	N	%	
T0-30	Maintained	26	92.9	25	92.6	0.970
	Improved 1	0	0.0	1	3.7	0.304
	Improved 2	2	7.1	1	3.7	0.574
T0-90	Maintained	26	92.9	25	92.6	0.970
	Improved 1	0	0.0	1	3.7	0.304
	Improved 2	2	7.1	1	3.7	0.574
T0-180	Worsened 1	1	3.6	2	7.4	0.531
	Maintained	10	35.7	18	66.7	0.022
	Improved 1	13	46.4	5	18.5	0.027
	Improved 2	4	14.3	2	7.4	0.413
T30-90	Maintained	28	100	27	100	- x -
T30-180	Worsened 1	1	3.6	2	7.4	0.531
	Maintained	12	42.9	20	74.1	0.019
	Improved 1	13	46.4	4	14.8	0.011
	Improved 2	2	7.1	1	3.7	0.574
T90-180	Worsened 1	1	3.6	2	7.4	0.531
	Maintained	12	42.9	20	74.1	0.019
	Improved 1	13	46.4	4	14.8	0.011
	Improved 2	2	7.1	1	3.7	0.574

We noticed a statistical significance in the T0-180, T30-180, and T90-180 delta values. Exemplifying the result in the delta from T0-180, we have statistical significance for results that held, in which we had 35.7% in the open surgery group and 66.7% in the Z-plasty group (p = 0.022). We also found significance for the change to the Improved 1 category, in which in the open surgery group the index was 46.4% compared with 18.5% of Z-plasty (p = 0.027).

## DISCUSSION

We analyzed the postoperative results of TCL enlargement by Z-plasty compared with the complete opening of the TCL.

TCL is a strong anatomical structure, whose main function is to serve as a pulley to keep the flexor tendons close to the center of rotation of the wrist.^5^ If these tendons move anteriorly during wrist flexion, this reduces their contraction force. After the division of TLC, many surgeons observe an anterior displacement of the flexor tendons and median nerve. In this study, the authors concluded that the complete division of TLC leads to reduced grip strength and prolonged palmar pain in the scar region.

According to the results of this study, the increase in grip strength was higher in patients in the group that TCL was completely opened, contrary to Lluch’s findings. ^10^

In a study with 52 patients and more than 25 weeks of follow-up, Dias et al. ^(^ ^11^ found no advantage over the technique of widening the TCL in Z-plasty over the complete opening. Recovery of neurological symptoms, functional results, and postoperative pain were evaluated. Similar results were obtained by Karlsson et al., ^(^ ^12^ in which 99 patients were evaluated in a retrospective study comparing the two techniques. Patients subjected to enlargement in Z-plasty of the TCL presented longer time away from work. According to the authors, the probable explanations were the longer immobilization time in the postoperative period of patients in the Z-plasty group as well as a more extensive exploration of the volar tissues, leading to a longer period of local inflammation. Thus, there is no advantage in rebuilding the TCL.

Dias et al., ^(^ ^11^ in a randomized double-blind study, compared the two techniques in 52 hands in a follow-up of 25 weeks. Function questionnaires were used to evaluate the severity of the disease. Their study showed no identifiable benefits in performing TCL enlargement for carpal tunnel decompression.

Castro-Menendéz et al., ^(^ ^13^ showed similar results in a study that evaluated 80 patients divided into two groups, a model similar to the previous study. In this study, the follow-up time was up to one year and no statistically significant differences were identified in the grip strength and presence of pillar pain between the two groups.

The grip strength was also evaluated by Karlsson et al. ^(^ ^12^ in a study in which the open technique for opening the TCL was compared with enlargement by Z-plasty. Patients subjected to TCL repair presented higher mean grip strength with the wrist at 45° extension, while the group subjected to the simple opening of the TCL had greater average grip strength when measured with the wrist at 45° of flexion.

Another study in which grip strength in patients who had undergone TCL reconstruction was higher than in patients who had undergone simple opening was that of Gutierrez-Monclus et al. ^(^ ^14^ A total of 177 patients were evaluated in a model similar to our study, being 59 patients subjected to stretching and 59 being the control group subjected to the simple opening of the TCL. The conclusion was that TCL reconstruction resulted in greater improvement of grip strength than in the group subjected to simple retinaculotomy.

In our study, the two techniques were equivalent when performance on the QuickDASH questionnaire was evaluated in isolation. Among the three criteria evaluated, this is the most subjective; however, it is the one that most relates to the patient’s perception regarding the improvement of symptoms and activities of daily living in the postoperative period. Regarding the sensitivity test by monofilaments, the group in which Z-plasty was used to widen the TCL showed greater recovery. In a similar study, Jakab, Ganos and Cook, ^(^ ^15^ performed the two-point discrimination test to evaluate the sensitivity of patients subjected to complete TCL opening compared with its enlargement by Z-plasty. Their study showed no relevant difference between the two techniques.

A commonly found complication in the literature related to the surgical opening of TCL is a pain in the thenar eminence region and in the region of the surgical scar, also called the “pillar pain”. Seitz and Lall^9^ relate this pain to a combination of the complete opening of the TCL with exposure of nerve endings and loss of the anatomical covering of the carpal tunnel that would have a biomechanical function, acting as a pulley for the flexor tendons, and a neuroprotective function. Their study failed to find a significant difference with six months of follow-up between the two groups. In a similar study, Saravi et al. ^(^ ^16^ concluded that although both groups showed improvement in pain and normal grip strength 12 weeks after surgery, the group subjected to Z-plasty showed a significant reduction in hand pain, shorter duration of this pain, and a shorter period to achieve normal grip strength.

Comparing enlargement in TCL Z-plasty with the simple opening, Xu, Huang and Hou^17^ obtained an excellent improvement in function and patient satisfaction in the Z-plasty group. The authors cite this method as a more effective alternative to the conventional method of surgery for the treatment of carpal tunnel syndrome. Castro-Menéndez et al. ^(^ ^13^ also compared these two techniques and showed no difference between the two groups regarding grip strength, pillar pain, and functional improvement assessed by a questionnaire. Thus, Z-plasty shows no identifiable benefits to reduce postoperative complications. In this study, as well as in most studies similar to ours, a recurrent and important bias is that the surgeries were performed by different surgeons. In the case of this study, this bias is not present, since all surgeries were performed by the same physician.

## CONCLUSION

With the comparison of the conventional technique with the complete opening of the TCL in relation to the technique of its enlargement using Z-plasty, we conclude that both are equivalent in terms of functional improvement of the hand using QuickDASH as a questionnaire. This is the most subjective parameter that considers the patient’s perception of their symptoms, limitations for activities of daily living, and how much the disease interferes with their routine.

The group subjected to complete opening of the TCL showed better progression of grip strength in relation to the group subjected to Z-plasty. This data contradicts the idea that the complete opening of the TCL would eliminate its pulley function for the flexor tendons, leading to reduced grip strength.

Regarding the sensitivity test using monofilaments, the Z-plasty group showed greater improvements than the other group.

Considering these parameters, we conclude that there is no significant advantage in extending the TCL through Z-plasty in relation to its simple opening.
